# Scientific breakthroughs in the COVID-19 era: embracing Ernst Mayr towards an epistemic renewal of Medicine

**DOI:** 10.31744/einstein_journal/2026RW1785

**Published:** 2025-12-04

**Authors:** Bruno Gualano, Marcus Sacrini Ayres Ferraz, Anderson Luis Nakano

**Affiliations:** 1 Universidade de São Paulo Center of Lifestyle Medicine Faculdade de Medicina São Paulo SP Brazil Center of Lifestyle Medicine, Faculdade de Medicina, Universidade de São Paulo, São Paulo, SP, Brazil.; 2 Universidade de São Paulo Letras e Ciências Humanas Faculdade de Filosofia São Paulo SP Brazil Faculdade de Filosofia, Letras e Ciências Humanas, Universidade de São Paulo, São Paulo, SP, Brazil.; 3 Pontifícia Universidade Católica de São Paulo São Paulo SP Brazil Pontifícia Universidade Católica de São Paulo, São Paulo, SP, Brazil.

**Keywords:** Science, Philosophy, Knowledge, Vaccines, COVID-19, Coronavirus infections

## Abstract

The present study examines the development of medical science during the COVID-19 pandemic in light of Ernst Mayr's critique of Thomas Kuhn's philosophy of scientific revolutions. Kuhn's model, which emphasizes paradigm shifts and disruptive changes in scientific practice, contrasts with the evolutionary and cumulative nature of biological sciences, as argued by Mayr. Revisiting Mayr's critique, we question the applicability of Kuhn's model to the COVID-19 pandemic by using vaccine development as a case study. We argue that the rapid advancements in vaccine technology, particularly mRNA-based vaccines, are better understood as the outcome of multiple independent micro-revolutions in scientific fields, such as genetics, pharmacology, and molecular biology. These disciplines have advanced steadily over decades, facilitating the accelerated response to the pandemic. Consequently, the rapid, effective, and game-changing development and implementation of mRNA vaccines represented a major breakthrough in medical science; their trajectory diverged from Kuhn's concepts of scientific revolution and normal science. Through this analysis, we propose rethinking of medical epistemology – one that acknowledges the asynchronous yet non-disruptive progress of the medical sciences. Such a framework not only provides a more accurate understanding of scientific evolution but also offers a valuable tool for addressing societal challenges, such as vaccine hesitancy and public trust in science.

*"The endeavor of a unification of the sciences is a search for a Fata Morgana*.

*Ernst Mayr, 2004, What makes Biology unique?, p.36^(4)^*

## INTRODUCTION

Philosophy of science addresses metaphysical, axiological, and epistemological issues.^(1)^ Among these, epistemology is of particular relevance to the present study, which critically examines the explanatory limitations of physicalist epistemological theories – specifically as articulated in Thomas Kuhn's conception of scientific development^(2)^ – when applied to the *praxis* of medical sciences.

A similar endeavor was undertaken by Ernst Mayr,^(3,4)^ who challenged the Kuhnian model of disruptive scientific revolutions by emphasizing the inherently cumulative character of the biological sciences. Against the backdrop of the Darwinian and neo-Darwinian paradigm shifts of the nineteenth and twentieth centuries, Mayr argued that these pivotal developments in biology did not conform to Kuhn's framework of scientific revolutions.

In this study, we revisit the central aspects of Mayr's critique of Kuhn's epistemology. Building on Mayr's arguments, we extend his critique to the development of medical science, with particular attention to the development of vaccines against SARS-CoV-2, especially mRNA-based vaccines. This achievement is widely regarded as a revolutionary milestone in medical history and a decisive factor in overcoming one of humanity's most devastating pandemics. Nevertheless, it falls outside the principles of Kuhn's model of scientific revolution.

Finally, we conclude with a reflection on the necessity of advancing an epistemology of medical science that aligns with its ontology, evolution, and *praxis*. Such an epistemological framework would not only address the limitations of physicalist theories but also serve as a robust countermeasure against unfounded claims propagated by science denial movements, particularly those opposing vaccination.

### A Biologist's Critique of Kuhn's Philosophy of Science

*Thomas Kuhn's Structure of Scientific Revolutions* is widely regarded as one of the most influential works in the philosophy of science and serves as a foundational epistemological reference across scientific disciplines.

In brief, Kuhn proposed a dynamic structure for the development of science, comprising the pre-paradigmatic phase, normal science, crisis, revolution, and a subsequent phase of new normal science, continuing cyclically. The pre-paradigmatic phase, characterized by significant divergence, is a period in which no shared framework exists to guide scientific inquiry. During this stage, scientists debate which phenomena to study and which theoretical principles, methodologies, and values to adopt, as the field has not yet achieved mature scientific status.

The establishment of a paradigm transitions the field into a phase of normal science. According to Kuhn, a paradigm encompasses shared theoretical commitments, methods, and practices that define a scientific community's approach to problem-solving. Normal science involves solving "puzzles" within the boundaries of the paradigm, refining its theoretical framework, and addressing anomalies as they arise. Kuhn^(2)^ views this cumulative progress as relevant but not the only form of scientific advancement:

*"Normal science does not aim at novelties of fact or theory, and, when successful, finds none."*

*– Kuhn, T. (1970). The Structure of Scientific Revolutions (p.52).* ^(2)^

Nevertheless, scientific progress often requires the rupture that occurs during a crisis, a state in which the accumulation of unresolved anomalies undermines the adequacy of the prevailing paradigm. This can lead to a scientific revolution in which one paradigm is replaced by a new, incommensurable paradigm. Kuhn describes this shift as a revolution that transforms both science and perception:

*"(…) the successive transition from one paradigm to another via revolution is the usual developmental pattern of mature science."*

*– Kuhn, T. (1970). The Structure of Scientific Revolutions (p.12).* ^(2)^

Kuhn's emphasis on the discontinuity of scientific revolutions challenges traditional inductivist and falsificationist methodologies and invites debate regarding their applicability beyond the physical sciences.

Ernst Mayr, a German biologist renowned for his contributions to the theory of evolutionary synthesis,^(5)^ presented substantial critiques of Kuhn's epistemology throughout his prolific career. Mayr's divergence from physicalist theories of science motivated his pursuit of an epistemology tailored to the unique nature of biology.

Mayr's epistemology was grounded in Darwinism, a collection of theories that evolved and became interwoven into the modern corpus of biological science.^(3-5)^ In contrast to Kuhn, Mayr argued that while the Darwinian revolution reached its zenith with the publication of The Origin of Species,^(6)^ it was the culmination of numerous scientific micro-revolutions. These microrevolutions, which began independently in the eighteenth century, converged to produce a cohesive and robust theoretical framework for understanding life.

The consolidation of what is recognized as the Darwinian revolution required at least two and a half centuries of sustained scientific effort to replace the prevailing concepts and beliefs. Mayr identified six key components of this transformative process, summarized by Levit et al.^(7)^ as follows:

*"First, the biblical idea that the Earth is 6,000 years old was definitely refuted; second, both catastrophism and the concept of the steady-state world were abandoned and replaced by the idea of the slowly evolving world; third, the obligatory steady evolutionary progress toward perfection was replaced by adaptation and specialization; fourth, the idea of creation was bracketed and for explanatory purposes replaced by natural causes; fifth, essentialism was replaced by population thinking; and, finally, anthropocentrism was abolished and humans became part of the evolutionary stream. (…). All these "elements" in their entirety would, in any case, lead to a Darwinian-like theory, but if only some of them were adapted they would bring about alternative evolutionary theories (if evolutionary at all). (…) The Darwinian revolution was so slow, Mayr argued, because it required not merely the replacement of one scientific theory by another, but the rejection of at least the six basic beliefs listed above along with some methodological innovations [Mayr, 1972]. Darwin's seminal publication in 1859 was the midpoint of this gigantic, slow revolution, not its beginning nor end."*

As highlighted by Levit et al.,^(7)^ Mayr's epistemology of the life sciences, exemplified by the development of Darwinism, emphasizes the gradual and cumulative progression of knowledge. This advancement occurs through successive scientific micro-revolutions, leaving little room for delineating disruptive events or distinct periods of normal science, as posited by Kuhn.^(2)^

The asynchronous nature of Darwinism further underscores Mayr's divergence from Kuhn's. Foundational theories integral to Darwinism – such as population speciation, natural selection, and evolution by common descent – emerged and were consolidated at different times and under varying circumstances, often accompanied by scientific upheaval. Contrary to expectations of the Kuhnian framework, the Darwinian revolution of 1859 was not a definitive moment for establishing these theories. Rather, each theory achieved recognition and acceptance independently based on its scientific merits within its own temporal and geographical context.

As Mayr meticulously argued,^(4,5)^ the investigation of knowledge production in biology is not well supported by Kuhn's theoretical constructs. The history of biology reveals neither genuine paradigm shifts nor periods of normal science before or after the landmark Darwinian revolution of 1859. Furthermore, the presumed incommensurability of paradigms is rendered untenable by the coexistence of seemingly conflicting theories and asynchronous developmental trajectories.

In Mayr's view, the evolution of biological science is analogous to Darwinian processes: it proceeds through gradual, cumulative, and non-saltationist changes. These incremental advancements enable the "natural selection" of scientifically robust theories, fostering a steady and coherent progression of knowledge within the field.

This perspective holds value for modern medical sciences. These fields have evolved through the integration of knowledge from physical, chemical, social, and technological disciplines while remaining firmly grounded in the biological sciences. The development of coronavirus disease 2019 (COVID-19) vaccines serves as a prime example of how Mayr's concept of scientific advancement can be applied to medicine.

### Novel Vaccines to Overcome a Lethal Pandemic: A Case of "Revolution without Disruption" in Medical Sciences

COVID-19, caused by the SARS-CoV-2 virus, has had catastrophic consequences, claiming over 7 million lives globally, including more than 700,000 in Brazil. Approximately 11% of those infected develop long COVID, a syndrome characterized by more than 150 persistent and debilitating symptoms.^(8)^ The pandemic has overwhelmed healthcare systems worldwide, straining their capacity and exacerbating existing inequalities in healthcare delivery.

The socioeconomic and educational impacts have been equally profound. To limit viral transmission, governments suspended activities in non-essential sectors (*e.g.*, bars, restaurants, and gyms), closed schools, and adopted remote teaching. In countries such as Brazil,^(9)^ and the United States,^(10)^ marginalized groups – particularly Black and economically disadvantaged populations – suffered disproportionately. These groups experienced higher infection rates, elevated mortality, and greater social vulnerabilities, such as unemployment, food insecurity, and school dropout. This inequity has led some scholars to frame the COVID-19 crisis as a syndemic^(11)^ characterized by the interaction of health crises with social and economic inequalities.

Although social distancing and confinement measures were initially essential for controlling viral spread, their prolonged enforcement proved unsustainable, exacerbating economic, educational, and mental health challenges, particularly among vulnerable populations.^(9-11)^

In response to the mounting global health crisis, the urgency of developing effective interventions against the virus sparked unprecedented efforts in vaccine development. The rapid creation, testing, and approval of COVID-19 vaccines, particularly those based on mRNA technology, are often heralded as a scientific revolution. These vaccines significantly reduced infection rates and severe cases, resulting in a marked reduction in mortality. The return to normality in many societies is largely attributable to large-scale vaccination campaigns, which demonstrate a clear and favorable risk-benefit ratio.^(12,13)^ Serious adverse events associated with vaccination are rare, especially when compared with the much greater health risks of remaining unvaccinated.

Despite their extraordinary impact, the rapid and effective development of COVID-19 vaccines was not an isolated breakthrough but part of a broader, asynchronous continuum of scientific progress. Expedited vaccine timelines reflect longstanding trends in innovation, building on foundational advances in mRNA technology that began in the 1980s. These advances were rooted in early research on the molecular biology of mRNA and its therapeutic potential.^(14)^ Therefore, although scientific advances during the pandemic were unprecedented in scale and speed, and for that reason often described as revolutionary in public discourse, they do not meet the criteria of the Kuhnian scientific revolution, as will be detailed later.

The mRNA technology that enabled the creation of COVID-19 vaccines is the culmination of decades of dedicated research by Drew Weissman and Katalin Karikó, as described extensively elsewhere.^(15,16)^ Their journey was marked by both breakthroughs and setbacks, ultimately leading to transformative innovations that reshaped vaccine development. In the 1990s, Katalin Karikó, a Hungarian biochemist, became captivated by the therapeutic potential of mRNA.^(15,16)^ She envisioned it as a tool to instruct cells to produce specific proteins for therapeutic purposes, such as treating diseases or generating immunity. However, early attempts faced two major obstacles: the inherent instability of mRNA and inflammatory response of the immune system to synthetic mRNA.

Despite difficulties in securing funding and facing professional setbacks, Karikó joined the University of Pennsylvania, where she began collaborating with immunologist Drew Weissman in the late 1990s. This partnership proved to be a turning point in mRNA research. In 2005, Karikó and colleagues made a breakthrough discovery: modifying one of the mRNA nucleosides, pseudouridine, significantly reduced the immune response without compromising its effectiveness.^(17)^ This modification allowed synthetic mRNA to remain stable and functional within the body, overcoming a critical barrier to therapeutic use. This breakthrough opened new avenues for mRNA technology, making it feasible for safe applications in humans. Yet another challenge remained: delivering mRNA effectively into cells. This challenge was eventually overcome through the development of lipid nanoparticles (LNPs), protective fat-based carriers that shield mRNA during delivery and ensure successful uptake by target cells.

Despite their groundbreaking discoveries, Karikó and colleagues initially struggled to gain recognition and funding. Momentum shifted in the 2010s, when biotech companies such as Moderna and BioNTech began investing in mRNA technology, showing promise for vaccine development against infectious diseases and cancer. Before the COVID-19 pandemic, mRNA technology had demonstrated potential in addressing diseases such as influenza, Zika, and rabies, although no mRNA-based vaccine had yet reached the market. Key advancements during this period included optimizing mRNA stability, refining delivery systems such as LNPs, and enhancing immune responses.^(15,16)^ These incremental breakthroughs laid the foundation for the rapid development of COVID-19 vaccines.

When the SARS-CoV-2 genome was sequenced and shared publicly in January 2020. mRNA vaccine candidates were rapidly designed. This marked the beginning of an unprecedented global effort to combat the pandemic. The modular nature of mRNA vaccines facilitated this speed, enabling researchers to insert the genetic sequence of the spike protein into an established framework without starting from scratch. Preclinical studies began almost immediately, demonstrating promising safety and immunogenicity profiles. Human clinical trials progressed swiftly, with phases I, II, and III (typically conducted sequentially), often overlapping to accelerate timelines, while still adhering to rigorous safety and efficacy standards.

By mid-2020, both Pfizer-BioNTech and Moderna had entered late-stage clinical trials, enrolling tens of thousands of participants globally. These trials confirmed the vaccine's high efficacy (approximately 95%) in preventing symptomatic COVID-19 and documented their robust safety profiles. The regulatory process, traditionally a major bottleneck in vaccine development, was expedited through emergency use authorizations (EUAs). Regulatory agencies worldwide prioritized the COVID-19 vaccine review and conducted interim analyses as data emerged, enabling timely decisions without compromising scientific integrity. By December 2020, less than a year after the virus was identified, Pfizer-BioNTech and Moderna vaccines received EUAs, marking the first approval of mRNA-based vaccines for public use.^(15,16)^

Mass production and distribution posed additional challenges, requiring unparalleled coordination among governments, manufacturers, and healthcare systems. The scalability of mRNA technology was a crucial factor in meeting global demand. Because the platform relies on synthetic processes, rather than biological systems, it enabled rapid manufacturing. Cold-chain logistics, essential for maintaining mRNA vaccine stability, presented significant challenges but were overcome through innovative solutions and international cooperation. By mid-2021, billions of doses had been distributed, profoundly altering the course of the pandemic.^(15,16)^

Each milestone reflected a convergence of scientific innovation, regulatory flexibility, and global cooperation. While these timelines were unprecedented, they represented the culmination of decades of progress in vaccine research rather than a complete departure from historical trends.

Accordingly, the extraordinary development and implementation of mRNA vaccines diverged from Kuhn's view of scientific revolution in several aspects. Decades of research in molecular biology and biochemistry gradually resolved challenges, such as mRNA stability and immunogenicity without entering a state of crisis or rejecting existing paradigms. Advances in immunology – particularly in understanding immune responses to the spike protein target – proceeded incrementally. Similarly, the development of LNPs in chemical engineering illustrates the steady innovation within established frameworks. Even the atypical overlap of clinical trial phases reflected practical acceleration rather than a radical break from scientific norms.

This trajectory contrasts with Kuhn's notion of incommensurable paradigms, in which scientific progress occurs through a radical break with prior knowledge. In the case of mRNA vaccines, progress unfolded through interdisciplinary collaboration, integrating new knowledge without entirely discarding established practices. Although often described as revolutionary in mainstream narratives, their development differs from Kuhn's concept of scientific revolution. As Kuhn^(2)^ argues:

*"Scientific revolutions are here taken to be those non-cumulative developmental episodes in which an older paradigm is replaced in whole or in part by an incompatible new one."*

– *Kuhn, T. (1970) The Structure of Scientific Revolutions (p.92).*^(2)^

Unlike Kuhn's "non-cumulative" shifts, mRNA vaccine development exemplifies cumulative, collaborative innovation – building on existing scientific frameworks while simultaneously transforming medical science.

It is also misleading to classify these advances as normal science, given their profound social, economic, and medical impact. Although mRNA vaccine development shares certain features of normal science – incremental progress, refinement, and extension of existing knowledge – it does not fit neatly within a stable paradigm. As Kuhn proposed, normal science is not inherently novel but represents the steady extension of the paradigm's scope and precision.

This was not the case with mRNA vaccines, which introduced a conceptual and technological shift that went beyond incremental refinement. Thus, their emergence in response to COVID-19 occupies an ambiguous position within Kuhn's theory: neither a strict Kuhnian scientific revolution nor an example of normal science.

### Insights into the social efficacy of embracing Mayr in medical sciences

Despite the well-established benefits of COVID-19 vaccination, vaccine hesitancy has reached alarming levels, contributing to high global mortality rates. A significant factor driving this hesitancy is the enduring influence of the modern antivaccine movement, which can be traced back to a fraudulent 1998 study published in The Lancet.^(18)^ This study falsely linked autism to the MMR vaccine (measles, mumps, and rubella), and although it was retracted and discredited by the scientific community,^(19)^ anti-vaccine rhetoric continues to rely on these debunked claims.

Fear and doubt are the primary drivers of vaccine reluctance,^(20)^ which the World Health Organization (WHO) has identified as one of the top ten threats to global health.^(21)^ In the case of COVID-19 vaccines, the unprecedented speed of development has led many to question their safety.^(22,23)^ This skepticism is based on two intertwined concerns:

- The belief that vaccines created using new, untested technologies cannot be trusted for safety.
- The assumption that a rapid and improvised testing process undermines vaccine safety.

Both concerns are based on inaccurate premises. With regard to the first concern, particularly for mRNA vaccines, it is erroneous to claim that these technologies were untested. The development of mRNA vaccines was built upon decades of scientific advancements in molecular biology, immunology, nanotechnology, and genetic engineering, all of which are fundamental to medical research.^(23,24)^ Regarding the second concern, although the testing process was expedited to address the urgent health crisis, it adhered to the same rigorous standards historically established by the scientific community, ensuring that the quality of the evidence was not compromised. Comparative analyses of the development timelines show that COVID-19 vaccines were consistent with traditional vaccine development processes. They reached the conclusion that COVID-19 vaccines’ timelines are reasonably aligned with previous vaccine development experiences. In fact, COVID-19 vaccines required more time to develop than some commonly used drugs today, indicating that they were not "outliers."

In popular culture and non-expert domains, the concept of paradigm shifts is frequently misapplied, transforming scientific progress into a narrative of sudden, dramatic revolutions. Films, novels, and books often depict scientific breakthroughs as radical departures from prior knowledge, portraying science as a sequence of conflicts between old and new paradigms, in which established ideas are abruptly overturned. While this view may hold in certain fields, particularly those investigating nonliving systems and the physical universe, it distorts the perception of scientific progress in the medical field. Innovations such as COVID-19 vaccines are often misperceived as sudden or radical, even when they are rooted in decades of prior research. The public may view the introduction of new vaccines as a revolutionary break from the established scientific paradigm, creating uncertainty and raising concerns regarding their safety and efficacy. This misperception is further amplified by misinformation, sensationalist media narratives, and inappropriate application of scientific concepts, ultimately contributing to vaccine hesitancy and undermining public trust in medical interventions.

To counter this notion, historical evidence demonstrates that the emergence of mRNA-based COVID-19 vaccines did not represent a sudden revolutionary leap; rather, it was the culmination of decades of incremental, asynchronous advances. In our view, this perspective on scientific progress aligns more closely with Mayr's view of scientific development and contrasts with Kuhn's notion of a scientific revolution (Table 1).

**Table 1:** Kuhn's vs. Mayr's theories of scientific advances in light of mRNA COVID-19 vaccine development

Kuhn's Theory	Mayr's Counter-Theory	New COVID-19 vaccines
Science progresses through paradigm shifts, marked by disruptive revolutions	Science advances gradually through micro-revolutions and cumulative knowledge	The development of mRNA vaccines exemplifies incremental progress built on decades of research
Paradigms are incommensurable, leading to complete replacement during a revolution	Competing theories can coexist and contribute to a broader scientific understanding	mRNA technology coexists with traditional vaccine approaches, enhancing rather than replacing them
Revolutions occur when anomalies accumulate, rendering the current paradigm untenable	Incremental advancements refine and extend existing theories without abrupt breaks	mRNA vaccines represent the refinement of molecular biology, immunology, and nanotechnology

The social efficacy of adopting Mayr's perspective lies in its potential to reduce skepticism regarding medical science, particularly with respect to perceived disruptive innovations. By emphasizing the incremental and cumulative nature of scientific development, Mayr's approach clarifies that mRNA vaccines were not unexpected or untested innovations, but rather the result of years of foundational research. This perspective challenges the narrative that these vaccines were "rushed" or "emerged suddenly," clarifying that COVID-19 mRNA vaccines are the product of decades of rigorously tested, and refined scientific research.

## Concluding Remarks

The development of COVID-19 vaccines, particularly those based on mRNA technology, illustrates the power of cumulative scientific progress in medical science. These achievements, although extraordinary in speed and impact, reflect decades of incremental advancement, rather than a singular revolution.

Applying Kuhn's *stricto sensu* definition of a scientific revolution leads to two possible conclusions: (i) the unprecedented medical advances achieved during the pandemic constituted a paradigm disruption – an interpretation we consistently reject – or alternatively (ii) these advances were merely the outcome of Kuhnian normal science. The latter view, however, is also problematic, since the extraordinary breakthroughs of the pandemic do not align with Kuhn's definition of normal science, which "does not aim at novelties of fact or theory and, when successful, finds none."

Instead, we argue that these advances reflect a unique form of scientific progress, which may be described as micro-revolutions from Ernst Mayr's perspective. In this view, groundbreaking achievements occur without the paradigm disruptions envisioned by Kuhn. These developments conform neither to Kuhnian revolutions nor to normal science, but rather to a distinct mode of progress that has profoundly transformed medical science and impacted society at large.

We contend that Mayr's model provides a compelling framework to address skepticism and vaccine hesitancy. By presenting scientific advancements as the culmination of decades of research rather than abrupt or disruptive shifts, it is possible to foster greater public trust in medical innovations. One potential limitation of Mayr's framework is its tendency to understate the impact of rapid advancements in the field, such as the transition from the miasma theory to the germ theory, which might be interpreted as a more abrupt shift rather than a purely incremental progression. Moreover, while Mayr's model closely aligns with the evolutionary trajectory of biological sciences, its applicability to more physicalist fields – such as biophysics, medical engineering, and medical physics – may warrant further exploration. Notwithstanding these gaps, Mayr's perspective remains a robust epistemological lens for understanding the cumulative and evolutionary progress of medical sciences. These sciences advance through mechanisms that are neither entirely "revolutionary" nor completely "normal" in the Kuhnian sense. This mode of progress merits further philosophical consideration.

## Data Availability

The underlying content is contained within the manuscript.
